# Role of angiotensin-converting enzyme 2 (ACE2) in COVID-19

**DOI:** 10.1186/s13054-020-03120-0

**Published:** 2020-07-13

**Authors:** Wentao Ni, Xiuwen Yang, Deqing Yang, Jing Bao, Ran Li, Yongjiu Xiao, Chang Hou, Haibin Wang, Jie Liu, Donghong Yang, Yu Xu, Zhaolong Cao, Zhancheng Gao

**Affiliations:** 1 Department of Pulmonary and Critical Care Medicine, Peking University People’s Hospital, Beijing, China; 2 Department of Pulmonary and Critical Care Medicine, The Central Hospital of Wuhan, Tongji Medical College, Huazhong University of Science and Technology, Wuhan, China; 3 Division of Pulmonary Diseases, State Key Laboratory of Biotherapy of China, and Department of Respiratory and Critical Care Medicine, West China Hospital of Sichuan University, Chengdu, China; 4 Department of Emergency, The 940th Hospital of Joint Logistics Support Force of Chinese People’s Liberation Army, Lanzhou, China; 5 Department of Cardiology, Peking University People’s Hospital, Beijing, China; 6 Department of Endocrinology, The First Affiliated Hospital of Zhengzhou University, Zhengzhou, China; 7 Department of Vascular and Endovascular Surgery, Chinese PLA General Hospital, Beijing, China

**Keywords:** SARS-CoV-2, COVID-19, Angiotensin-converting enzyme 2, Multi-organ injury

## Abstract

**Abstract:**

An outbreak of pneumonia caused by severe acute respiratory syndrome coronavirus 2 (SARS-CoV-2) that started in Wuhan, China, at the end of 2019 has become a global pandemic. Both SARS-CoV-2 and SARS-CoV enter host cells via the angiotensin-converting enzyme 2 (ACE2) receptor, which is expressed in various human organs. We have reviewed previously published studies on SARS and recent studies on SARS-CoV-2 infection, named coronavirus disease 2019 (COVID-19) by the World Health Organization (WHO), confirming that many other organs besides the lungs are vulnerable to the virus. ACE2 catalyzes angiotensin II conversion to angiotensin-(1–7), and the ACE2/angiotensin-(1–7)/MAS axis counteracts the negative effects of the renin-angiotensin system (RAS), which plays important roles in maintaining the physiological and pathophysiological balance of the body. In addition to the direct viral effects and inflammatory and immune factors associated with COVID-19 pathogenesis, ACE2 downregulation and the imbalance between the RAS and ACE2/angiotensin-(1–7)/MAS after infection may also contribute to multiple organ injury in COVID-19. The SARS-CoV-2 spike glycoprotein, which binds to ACE2, is a potential target for developing specific drugs, antibodies, and vaccines. Restoring the balance between the RAS and ACE2/angiotensin-(1–7)/MAS may help attenuate organ injuries.

**Graphical abstract:**

SARS-CoV-2 enters lung cells via the ACE2 receptor. The cell-free and macrophage-phagocytosed virus can spread to other organs and infect ACE2-expressing cells at local sites, causing multi-organ injury. 
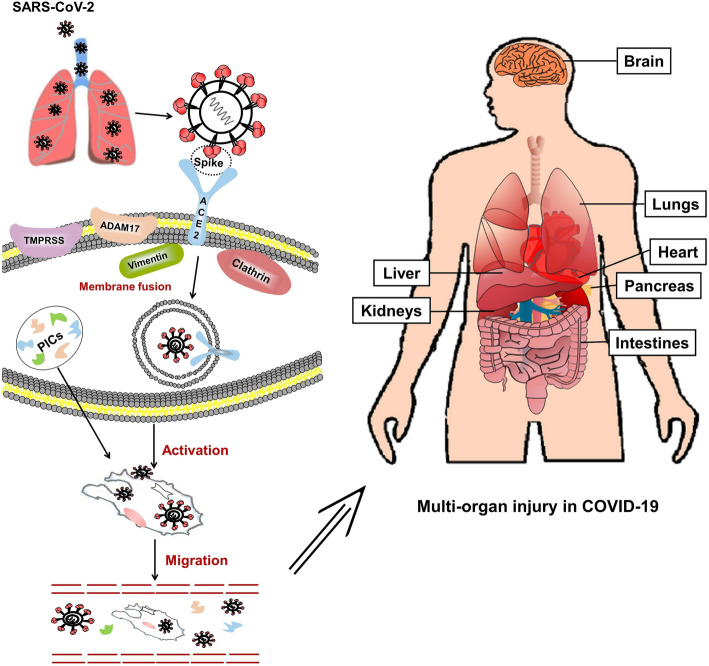

## Background

At the end of 2019, an outbreak of a novel coronavirus (2019-nCoV) was reported in Wuhan, Hubei Province, China [1, 2]. The outbreak has become a global pandemic. This virus seems to be much more contagious than severe acute respiratory syndrome (SARS) coronavirus (SARS-CoV) and Middle East Respiratory Syndrome (MERS) coronavirus (MERS-CoV). By the end of June 8, 2020, there have been more than 7,000,000 confirmed cases of coronavirus disease 2019 (COVID-19), with nearly 400,000 fatalities worldwide.

Full-length genome sequencing revealed that 2019-nCoV shares 79.5% sequence identity with SARS-CoV, and pairwise protein sequence analysis found that it belonged to the class of SARS-related coronaviruses [3]. Both 2019-nCoV and SARS-CoV enter host cell via the same receptor, angiotensin-converting enzyme 2 (ACE2) [3]. Therefore, this virus was subsequently renamed SARS-CoV-2. Although the overall mortality rate of COVID-19 caused by SARS-CoV-2 is lower than that of SARS and MERS, organ dysfunction, such as acute respiratory distress syndrome (ARDS), acute cardiac injury, acute hepatic injury, and acute kidney injury are quite common in severe cases. ACE2, a homolog of angiotensin-converting enzyme (ACE), which is expressed in a variety of human organs and tissues, has extensive biological activities and can counteract the negative role of the renin-angiotensin system (RAS) in many diseases [4–6]. Considering that the spike protein of SARS-CoV-2 interacts with ACE2, as does that of SARS-CoV, COVID-19 may have a pathogenic mechanism similar to that of SARS. In this review, we will discuss the role of ACE2 in COVID-19, and its potential therapeutic targets, aiming to provide more information on the management of the epidemic.

### RAS and ACE2

**Fig. 1:**
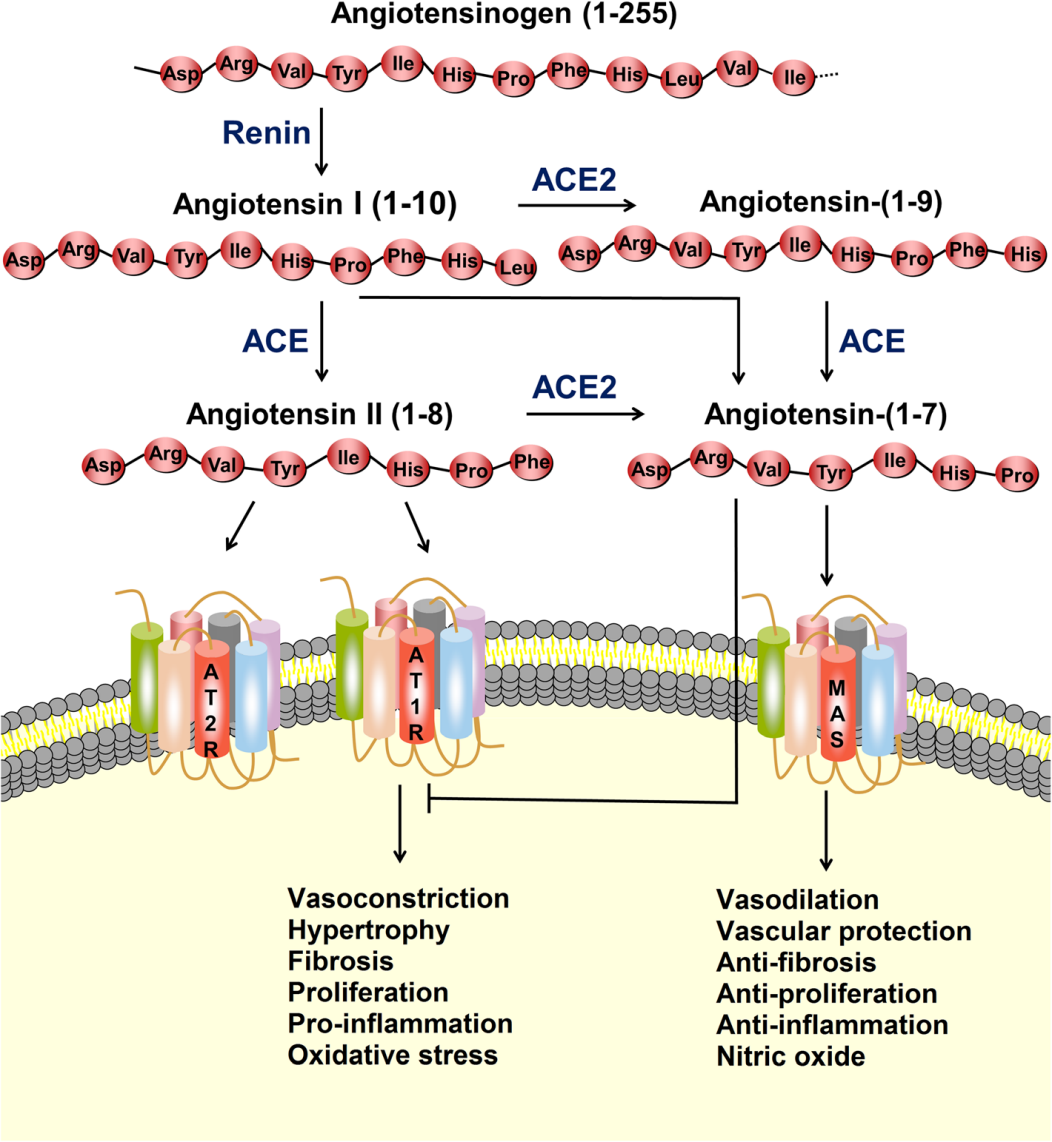
The renin-angiotensin system (RAS) and ACE2/angiotensin-(1–7)/MAS axis. The protease renin converts angiotensinogen to Ang-I, which is subsequently converted to Ang-II by angiotensin-converting enzyme (ACE). Ang-II can bind to the angiotensin type 1 receptor (AT1R) to exert actions, such as vasoconstriction, hypertrophy, fibrosis, proliferation, inflammation, and oxidative stress. ACE2 can covert Ang-I and Ang-II to angiotensin-(1–7). Angiotensin-(1–7) binds to the MAS receptor to exert actions of vasodilation, vascular protection, anti-fibrosis, anti-proliferation, and anti-inflammation. Ang-II can also bind to the angiotensin type 2 receptor (AT2R) to counteract the aforementioned effects mediated by AT1R

The RAS is a complex network that plays an important role in maintaining blood pressure as well as electrolyte and fluid homeostasis, affecting the function of many organs, such as the heart, blood vessels, and kidneys [6]. Angiotensin II (Ang-II), which is the most representative bioactive peptide in the RAS, widely participates in the progression of cardiovascular diseases, such as hypertension, myocardial infarction, and heart failure [7]. In the classic RAS, renin cleaves the substrate angiotensinogen to form the decapeptide angiotensin I (Ang-I), and then, ACE removes two amino acids at the carboxyl terminus of Ang-I to yield Ang-II (Fig. 1). To date, three Ang-II receptors have been identified, and the affinities of these receptors for Ang-II are similar, in the nanomolar range [7]. Among these receptors, angiotensin type 1 receptor (AT1R) binds to Ang-II, causing vasoconstriction, cell proliferation, inflammatory responses, blood coagulation, and extracellular matrix remodeling, whereas angiotensin type 2 receptor (AT2R) counteracts the aforementioned effects mediated by AT1R [8].

In 2000, two independent research groups discovered ACE2, a homolog of ACE, which can remove the carboxy-terminal phenylalanine in Ang-II to form the heptapeptide angiotensin-(1–7) [4, 9]. In addition, under the alternating effects of ACE2 and ACE, angiotensin-(1–7) can be formed without Ang-II (Fig. 1). In this metabolic pathway, Ang-I is first hydrolyzed by ACE2 to form angiotensin-(1–9), and angiotensin-(1–9) is then hydrolyzed by ACE to form angiotensin-(1–7). Ang-I can also be directly converted to angiotensin-(1–7) by endopeptidases and oligopeptidases [6]. Because of the higher affinity between ACE and Ang-I, the classical pathway of Ang-II to angiotensin-(1–7) is more common [6]. Angiotensin-(1–7), as a ligand, binds to the G-protein-coupled receptor MAS, which produces the opposite effect to that of Ang-II, and exerts a range of functions in multiple organs/systems [5, 6]. In addition to catalyzing the production of angiotensin-(1–7), ACE2 is involved in the uptake of amino acids in intestinal epithelial cells [10].

### ACE2 mediates SARS-CoV-2 infection

**Fig. 2:**
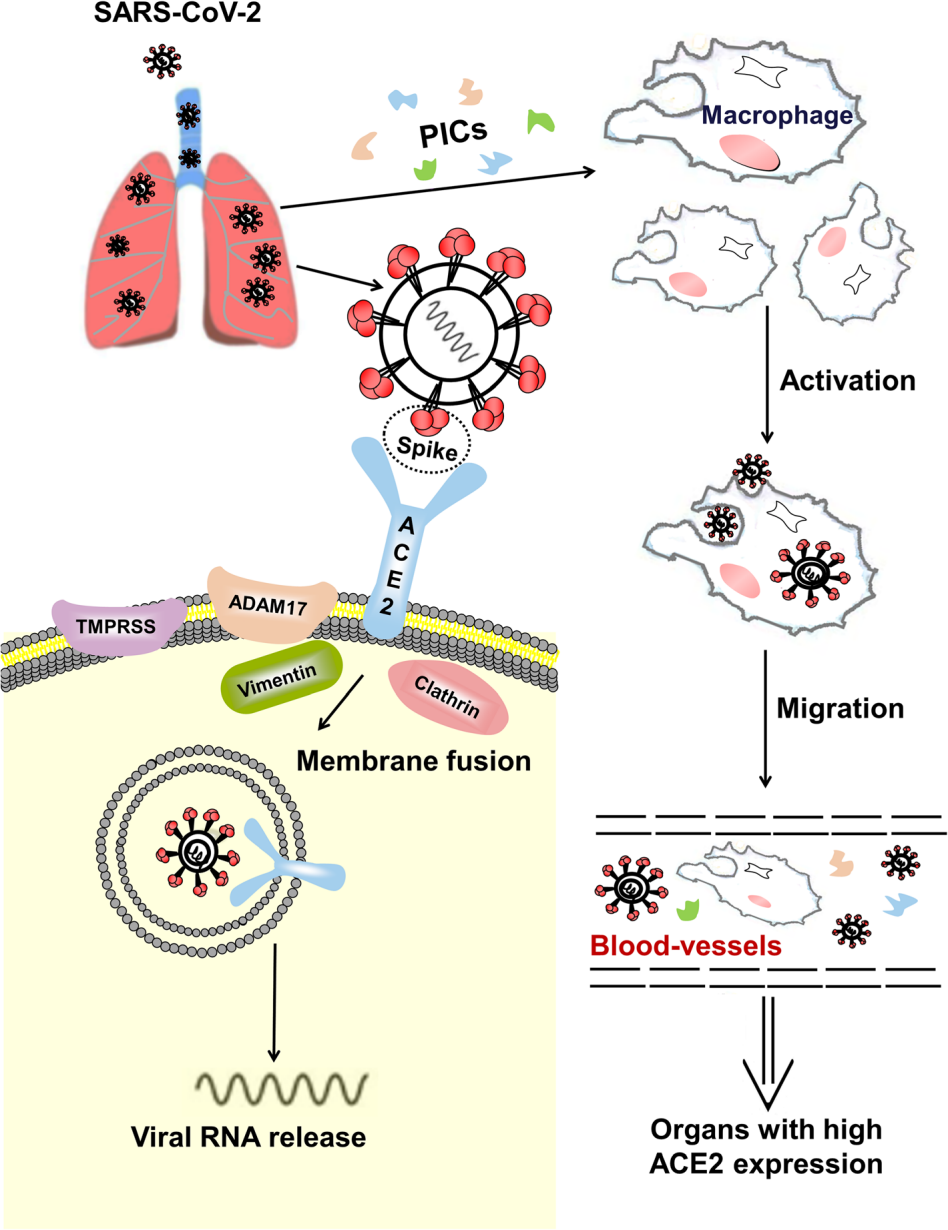
A model for the process of SARS-CoV-2 entering host cells in the lungs and attacking other organs. SARS-CoV-2 enters the lungs, where the spike glycoprotein of the virus binds to ACE2 on cells, allowing the virus enter the cells. Some transmembrane proteinases, such as transmembrane protease serine 2 (TMPRSS2) and a disintegrin metallopeptidase domain 17 (ADAM17) also participate in this process. For example, SARS-CoV-2 can use TMPRSS2 for spike protein priming in cell lines. The infected cells and inflammatory cells stimulated by viral antigens can produce pro-inflammatory cytokines (PICs) and chemokines to activate immunological reactions and inflammatory responses to combat the viruses. Cell-free and macrophage-phagocytosed viruses in the blood can be transmitted to other organs and infect ACE2-expressing cells at local sites

Entry into host cells is the first step of viral infection. A spike glycoprotein on the viral envelope of the coronavirus can bind to specific receptors on the membrane of host cells. Previous studies have shown that ACE2 is a specific functional receptor for SARS-CoV [11]. Zhou et al. showed that SARS-CoV-2 can enter ACE2-expressing cells, but not cells without ACE2 or cells expressing other coronavirus receptors, such as aminopeptidase N and dipeptidyl peptidase 4 (DPP4), confirming that ACE2 is the cell receptor for SARS-CoV-2 [3]. Further studies showed that the binding affinity of the SARS-CoV-2 spike glycoprotein to ACE2 is 10- to 20-fold higher than that of SARS-CoV to ACE2 [12]. The probable mechanism of SARS-CoV-2 entry into host cells based on SARS-CoV studies is displayed in Fig. 2. Briefly, the receptor-binding domain of the spike glycoprotein binds to the tip of subdomain I of ACE2 [11–14]. Membrane fusion of the virus and the host cell is activated after binding, and viral RNA is subsequently released into the cytoplasm, establishing infection. For SARS-CoV infection, intact ACE2 or its transmembrane domain is internalized together with the virus [15]. The catalytically active site of ACE2 is not occluded by the spike glycoprotein, and the binding process is independent of the peptidase activity of ACE2 [14]. Some transmembrane proteinases (such as a disintegrin and metallopeptidase domain 17 [ADAM17], transmembrane protease serine 2 [TMPRSS2], and TNF-converting enzyme) and proteins (such as vimentin and clathrin) may be involved in the binding and membrane fusion processes [16–21]. For example, ADAM17 can cleave ACE2 to cause ectodomain shedding, and TMPRSS2 can cleave ACE2 to promote viral uptake [16, 17].

ACE2 is expressed in nearly all human organs in varying degrees. In the respiratory system, the traditional immunohistochemical method and recently introduced single-cell RNA-seq analysis revealed that ACE2 is mainly expressed on type II alveolar epithelial cells, but weakly expressed on the surface of epithelial cells in the oral and nasal mucosa and nasopharynx, indicating that the lungs are the primary target of SARS-CoV-2 [22, 23]. Moreover, ACE2 is highly expressed on myocardial cells, proximal tubule cells of the kidney, and bladder urothelial cells, and is abundantly expressed on the enterocytes of the small intestine, especially in the ileum [22–24]. The cell-free and macrophage phagocytosis-associated virus may spread from the lungs to other organs with high ACE2 expression through blood circulation (Fig. 2). For example, up to 67% of patients who developed diarrhea during the course of SARS and quite a number of patients with COVID-19 showed enteric symptoms [25–27]. Active viral replication in enterocytes of the small intestine has been reported, and SARS-CoV-2 has been successfully isolated from fecal specimens [28, 29].

### ACE2 is associated with multi-organ injury in COVID-19

Autopsies of SARS patients showed that SARS-CoV infection can cause injury to multiple organs, such as the heart, kidney, liver, skeletal muscle, central nervous system, and adrenal and thyroid glands, besides the lungs [30, 31]. Most critically ill patients with COVID-19 also had multiple organ damage, including acute lung injury, acute kidney injury, cardiac injury, liver dysfunction, and pneumothorax [32]. As with SARS and COVID-19, organ injury is also frequently observed in MERS, especially the gastrointestinal tract and kidneys, while the incidence of acute cardiac injury is less common [33–36]. Unlike SARS-CoV and SARS-CoV-2, MESR-CoV uses DPP4 as its entry receptor, which is mainly expressed on pneumocytes, multinucleated epithelial cells, and bronchial submucosal gland cells of the lungs; epithelial cells of the kidney and small intestine; and activated leukocytes [37–39]. DPP4 is not abundantly expressed on myocardial cells [37–39]. Therefore, this indicates that organ involvement and injury is strongly associated with receptor distribution in the body.

According to the results of a series of studies on SARS, the pathogenesis of COVID-19 should be complex. The virus-induced inflammatory responses, including the excessive expression of cytokines and chemokines, excessive recruitment of inflammatory cells, insufficient interferon response, and possible production of auto-antibodies are deemed to be vital factors in disease pathogenesis [30]. Pro-inflammatory cytokines (PICs) and chemokines in plasma, such as interleukin (IL)-1, IL-6, IL-12, IL-8, monocyte chemoattractant protein-1 (MCP-1), and interferon-gamma-inducible protein 10 (IP-10), are significantly elevated in the plasma of patients with SARS [40, 41]. Significantly increased plasma concentrations of these PICs were also found in severe patients with COVID-19 [1]. Autopsy studies of SARS patients further found that PICs and MCP-1 were highly expressed in SARS-CoV-infected ACE2+ cells, but not in tissues without infected ACE2+ cells, suggesting virus-induced local immune-mediated damage [42].

In addition to acting as the receptor for SARS-CoV and SARS-CoV-2, ACE2 hydrolyzes Ang-II to angiotensin-(1–7), and the ACE2/angiotensin-(1–7)/MAS counteracts the negative effects of the RAS and exerts anti-inflammatory effects [6, 43]. Several studies have shown that SARS-CoV infection can downregulate ACE2 expression on cells, thereby disrupting the physiological balance between ACE/ACE2 and Ang-II/angiotensin-(1–7) and subsequently causing severe organ injury [44–47]. Given that SARS-CoV-2 is a species of SARS-related coronaviruses and uses ACE2 as its receptor, the downregulation of ACE2 expression may be involved in multiple organ injury in COVID-19.

**Fig. 3:**
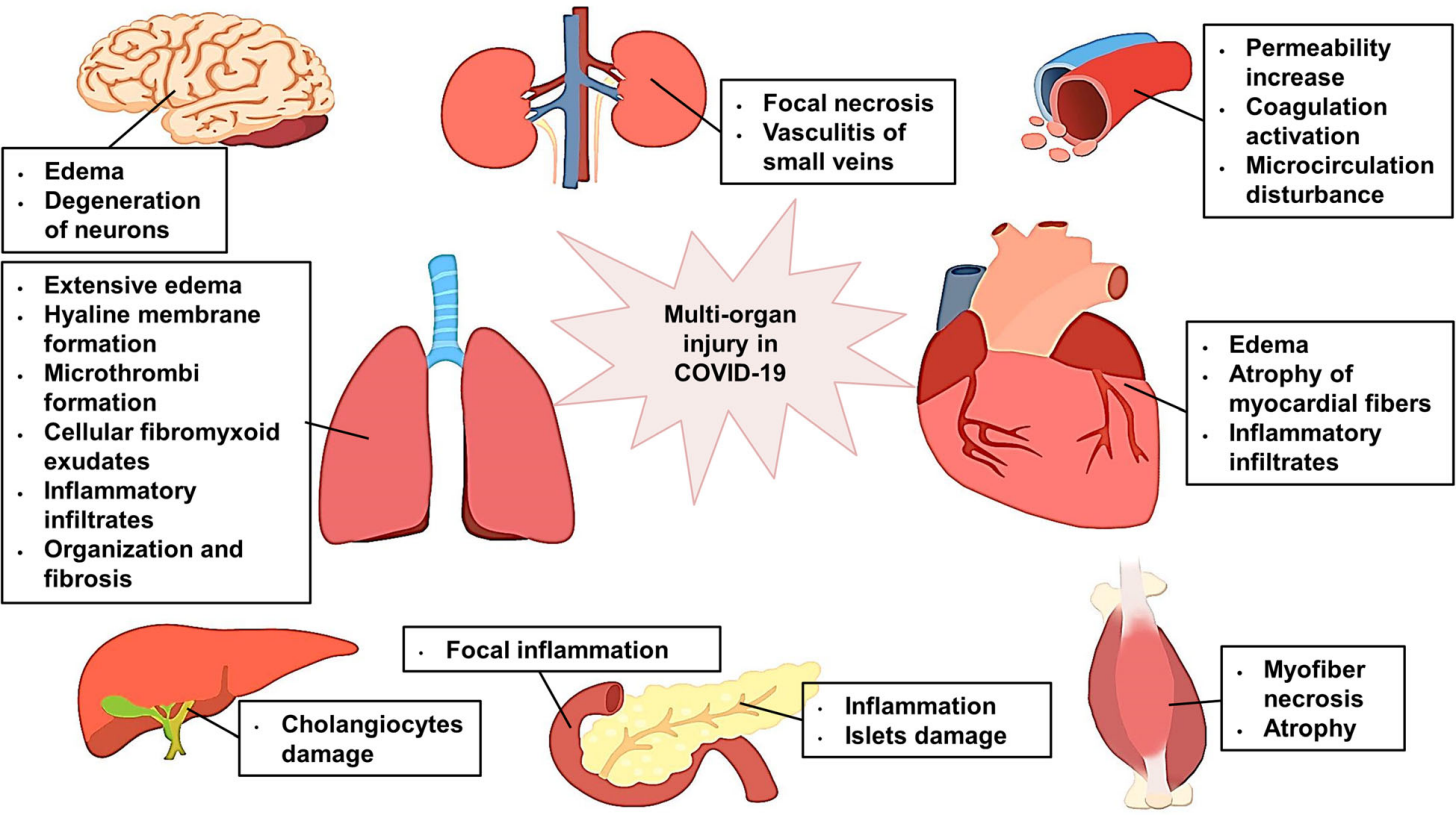
Main organs involved in COVID-19

Based on previous studies on SARS and recent studies on SARS-CoV-2, the multiple organ injury in COVID-19 (Fig. 3) and the possible role of ACE2 in organ injury are described below.

### Acute lung injury

Although the mortality rate in COVID-19 is lower than that in SARS and MERS, numerous patients have acute lung injury (ALI) after infection [26, 32]. Similar to the pathological features of SARS and MERS, severe diffuse alveolar damage, such as extensive edema, hyaline membrane formation, inflammatory infiltrates, microthrombi formation, organization, and fibrosis, was also observed in COVID-19, but with more cellular fibromyxoid exudates in the alveoli and small airways [48, 49]. The role of the RAS and ACE2 in ARDS/ALI has drawn great attention since the outbreak of SARS in 2003. Clinical studies have found that ACE insertion/deletion polymorphism may be correlated with the severity of ARDS [50, 51]. High Ang-II levels in the lungs can increase vascular permeability and cause pulmonary edema [52, 53]. Several studies have revealed the protective effects of the ACE2/angiotensin-(1–7)/MAS axis in the lungs. It alleviates lung inflammation, fibrosis, and pulmonary arterial hypertension, as well as inhibiting cancer cell growth, tumor angiogenesis, and tumor metastasis [6, 54–56]. In different animal models of ALI, ACE2-knockout mice exhibited enhanced vascular permeability, increased lung edema, neutrophil accumulation, and marked worsening of lung function compared with wild-type control mice [56]. Injection of recombinant human ACE2 protein or AT1R blockers into ACE2-knockout mice could decrease the degree of ALI [56].

SARS-CoV infection considerably reduces ACE2 expression in mouse lungs [46]. Further experiments showed that mere binding of recombinant SARS-CoV spike-Fc to human and mouse ACE2 could result in the downregulation of cell-surface ACE2 expression [46]. The spike-Fc protein worsened acid-induced ALI in wild-type mice, but did not affect the severity of lung failure in ACE2-knockout mice, indicating that the effect of spike protein on ALI is ACE2-specific [46]. Studies on influenza also found that ACE2 was significantly downregulated after H1N1 infection [57]. ACE2 deficiency significantly worsened the pathogenesis in infected mice, and inhibition of AT1 alleviated the severity of influenza H7N9 virus-induced lung injury [58, 59]. Moreover, Ang-II levels were elevated in H5N1- and H7N9-infected patients, which was associated with the severity of lung injury and predicted fatal outcomes in H7N9-infected patients [59, 60]. In patients with COVID-19, plasma Ang-II levels were markedly elevated and linearly associated with viral load and lung injury [61]. All these findings suggest that the RAS and ACE2 downregulation contribute to the pathogenesis of lung injury in COVID-19.

### Acute cardiac injury

The heart abundantly expresses ACE2, indicating that it is vulnerable to SARS-Cov-2 infection. Autopsies of patients with SARS revealed that 35% of them (7 of 20) were positive for the SARS-CoV genome in cardiac tissue, and patients with SARS-CoV cardiac infections had a more aggressive illness and earlier mortality than those without [47]. Edema of the myocardial stroma, inflammatory cell infiltration, and atrophy of cardiac muscle fibers was observed in patients with SARS and myocardial damage [30, 31, 62–64]. Cardiac injury is quite common among severely ill patients with COVID-19, and we found that early acute myocardial injury was associated with a higher risk of mortality [65].

The beneficial role of the ACE2/angiotensin-(1–7)/MAS axis in the heart has been well demonstrated [6]. It can induce vasorelaxation of coronary vessels, inhibit oxidative stress, attenuate pathological cardiac remodeling, and improve postischemic heart function [66]. ACE2 expression usually increases at the initial stage of heart injury, but decreases as the disease progresses [7]. ACE2 knockout in mice results in myocardial hypertrophy and interstitial fibrosis and accelerates heart failure [67, 68]. In addition, ACE2 knockout in mice aggravates cardiac dysfunction caused by diabetes [69]. In both SARS-CoV-infected mice and humans, ACE2 expression in myocardial cells is markedly downregulated in the heart [47]. According to recent studies [26, 70] and our data [65], a substantial number of patients with severe disease have hypertension as a comorbidity. Over-activation of the RAS may have already occurred in these individuals before infection. The significant downregulation of ACE2 and upregulation of Ang-II in COVID-19 results in RAS over-activation, and loss of the protective effects of angiotensin-(1–7) may aggravate and perpetuate cardiac injuries.

### Digestive system injury

The gastrointestinal tract, especially the intestine, is vulnerable to SARS-CoV and SARS-CoV-2 infections. SARS-CoV particles have been detected in epithelial cells of the intestinal mucosa, but not in the esophagus and stomach [30, 42]. The main pathological finding in the intestines of patients with SARS was the depletion of mucosal lymphoid tissue [71]. Only mild focal inflammation was detected in the gastrointestinal tract [71]. These findings may explain why gastrointestinal manifestations in COVID-19 are not severe and are transient.

Many patients with COVID-19 show a slight to moderate increase in serum levels of alanine aminotransferase (ALT) and/or aspartate aminotransferase (AST) during the course of infection [26, 72]. Autopsies of SARS patients revealed fatty degeneration, hepatocyte necrosis, and cellular infiltration in the liver [30]. However, SARS-CoV was not detected in the hepatic tissue of most patients autopsied [30]. Both immunohistochemistry and single-cell RNA-seq analyses showed that hepatocytes, Kupffer cells, and the endothelial lining of the sinusoids were negative for ACE2; only cholangiocytes were positive for ACE2 [22, 23, 73]. Gamma-glutamyl transpeptidase (GGT), which reflects cholangiocyte damage, was elevated in some COVID-19 patients [74]. These findings indicate that most acute hepatic injury may not be due to virus infection, but is highly likely due to other causes, such as drug hepatotoxicity, hypoxia, and systemic inflammation. Whether SARS-CoV-2 causes damage to the bile ducts by binding with ACE2 on cholangiocytes requires further investigation.

### Acute kidney injury

ACE2 is highly expressed in the kidney, especially in the apical membranes of proximal tubular epithelial cells, suggesting that the kidney is another target of SARS-CoV-2 [22, 23, 75]. Moreover, an imbalance between Ang-II and angiotensin-(1–7) caused by ACE2 deficiency may aggravate the vulnerability of the kidney to other factors causing acute kidney injury (AKI) [76]. SARS-CoV was detected in epithelial cells of the distal tubules, and viral sequences were identified in urinary samples from some patients [77, 78]. SARS-CoV-2 has also been isolated from urinary samples [79]. A retrospective analysis of 536 SARS patients showed that 6.7% of patients developed acute renal impairment during the course of the disease [80]. A large cohort study from New York showed that the incidence of AKI among patients with COVID-19 could reach 36.6% [81].

### Other organ and tissue injuries

#### Pancreas

Pancreatic cells highly express ACE2, indicating that COVID-19 may affect the pancreas [82]. It has been reported that up to 16% of patients with severe COVID-19 have elevated serum amylase and lipase levels, with 7% displaying accompanying significant pancreatic changes on CT scans [83]. Clinical presentation of acute pancreatitis has been reported in patients with COVID-19 [84]. ACE2/angiotensin-(1–7) plays a protective role in diabetes by improving pancreatic β cell survival, stimulating insulin secretion, and reducing insulin resistance [6]. Studies have shown that, compared to patients with non-SARS pneumonia, many more SARS patients who had no previous diabetes and had not received steroid treatment developed insulin-dependent acute diabetes during hospitalization [85, 86]. Moreover, plasma glucose levels and diabetes are independent predictors of mortality in patients with SARS [86]. Autopsies of some SARS patients found atrophy and amyloid degeneration in most pancreatic islets, suggesting the virus causes damage to the islets [64]. Therefore, COVID-19 may also influence pancreatic function, similar to SARS, and glucose levels should be closely monitored, especially in patients with diabetes or glucocorticoid treatment.

#### Skeletal muscles

Muscle weakness and elevated serum creatine kinase (CK) levels were observed in more than 30% of patients with SARS [87]. Mildly to moderately elevated CK levels were also observed in patients with COVID-19 on admission [88]. Myofiber necrosis and atrophy were observed in skeletal muscle tissues, but no SARS-CoV particles were detected by electron microscopy [30, 89]. Recent studies revealed that the RAS plays an important role in the pathogenesis of various skeletal muscle disorders, and the ACE2/angiotensin-(1–7)/MAS axis exerts protective effects against muscle atrophy [6]. Nevertheless, whether SARS-CoV-2 attacks the muscles and whether the downregulation of ACE2 is associated with myopathy is unclear.

#### Central nervous system

ACE2 is widely present in the brain, predominantly in neurons, and participates in the neural regulation of broad physiological functions, such as cardiovascular and metabolic activities, stress response, and neurogenesis [6, 90, 91]. In a mouse model, SARS-CoV invaded the brain through the olfactory bulb and then spread transneuronally to other areas [92]. Olfactory and gustatory dysfunctions have been reported in many patients with COVID-19, suggesting the involvement of the olfactory bulb in SARS-CoV-2 infection [93, 94]. SARS-CoV was isolated from human brain tissue specimens [31, 95]. Autopsies showed edema and focal degeneration of neurons in the brains of patients with SARS [30, 31]. Many patients (78/214) had neurologic manifestations in COVID-19, and SARS-CoV-2 was detected in the cerebrospinal fluid of a patient with encephalitis [96, 97]. Considering that SARS-CoV-2 has a much higher affinity for its receptor (ACE2) than SARS-CoV, the former could be capable of infecting and damaging the central nervous system.

#### Blood vessels

ACE2 is also expressed in the endothelial cells of small and large blood vessels, and the vascular endothelium can produce angiotensin-(1–7) [6, 22]. The ACE2/angiotensin-(1–7)/MAS axis induces vasodilatory, antiproliferative, and antithrombotic effects in the vasculature [6]. SARS RNA can be detected in the endothelia of the small veins in many tissues [98]. Plasma D-dimer levels are significantly elevated in severely ill patients with COVID-19 [1, 32, 72], and the occurrence of disseminated intravascular coagulation (DIC) at the early stage of the disease is not rare. Viral infection and inflammatory responses damage the integrity of the vascular endothelium, causing increased permeability, coagulation activation, and microcirculation disturbances, which may contribute to organ injury in COVID-19.

### Potential targets and drugs

As ACE2 is the receptor for both SARS-CoV and SARS-CoV-2, and some transmembrane proteinases such as ADAM17 and TMPRSS are involved in binding and membrane fusion processes, these sites may be potential targets in the development of antiviral drugs for COVID-19 treatment. For example, serum samples from patients with convalescent SARS can neutralize spike-driven entry of SARS-CoV-2 into host cells, suggesting that vaccines targeting the spike protein will be promising [18]. Studies have found that SARS-CoV-specific monoclonal antibodies and recombinant ACE2-Ig can potently neutralize SARS-CoV-2, and a hexapeptide of the receptor-binding domain of the spike protein binds to ACE2, thus blocking SARS-CoV entry [18, 99–101].

The downregulation of ACE2 in organs after virus infection disturbs the local balance between the RAS and ACE2/angiotensin-(1–7)/MAS axis, which may be associated with organ injuries. Animal studies have found that ACE inhibitor (ACEI) therapy can increase plasma angiotensin-(1–7) levels, decrease plasma Ang-II levels, and increase cardiac ACE2 expression, whereas angiotensin II receptor blockers (ARBs) can increase the plasma levels of both Ang-II and angiotensin-(1–7) as well as the cardiac expression and activity of ACE2 [102]. Thus, the use of ACEIs/ARBs, renin inhibitors, and angiotensin-(1–7) analogs may attenuate organ injuries by blocking the renin-angiotensin pathway and/or increasing angiotensin-(1–7) levels [103]. Other animal studies showed that ALI mediated by SARS-CoV spike or the influenza virus in mice could be rescued by the use of ARBs [46, 60, 104]. In a population-based study, the application of ACEIs and ARBs significantly reduced the 30-day mortality rate of patients with pneumonia requiring hospitalization [105]. There are also concerns that treatment with ACEIs/ARBs may facilitate infection and increase the risk of developing severe and fatal COVID-19 by increasing ACE2 expression levels in target organs [106]. However, two large cohort studies showed that ACEIs/ARBs use was not associated with increased SARS-CoV-2 infection, but was associated with a lower risk of all-cause mortality in hospitalized patients [107, 108]. Further studies are needed to test the protective effects of ACEIs/ARBs in COVID-19.

## Conclusions

The RAS and ACE2/angiotensin-(1–7)/MAS axis play important roles in various physiological and pathophysiological contexts. Both SARS-CoV-2 and SARS-CoV use ACE2 as the receptor for entry into host cells. Because ACE2 is highly expressed in various organs and tissues, SARS-CoV-2 not only invades the lungs but also attacks other organs with high ACE2 expression. The pathogenesis of COVID-19 is highly complex, with multiple factors involved. In addition to the direct viral effects and inflammatory and immune factors, the downregulation of ACE2 and imbalance between the RAS and ACE2/angiotensin-(1–7)/MAS axis may also contribute to the multiple organ injuries in COVID-19. The spike glycoprotein of SARS-CoV-2 is a potential target for the development of specific drugs, antibodies, and vaccines. Restoring the balance between the RAS and ACE2/angiotensin-(1–7)/MAS may help attenuate organ injuries in COVID-19.

## Data Availability

All data generated and/or analyzed during this study are included in this published article.

## Abbreviations

COVID-19: Coronavirus disease 2019
SARS: Severe acute respiratory syndrome
SARS-CoV-2: Severe acute respiratory syndrome coronavirus 2
ACE2: Angiotensin-converting enzyme 2
RAS: Renin-angiotensin system
MERS: Middle East Respiratory Syndrome
Ang-II: Angiotensin II
AT1R: Angiotensin type 1 receptor
AT2R: Angiotensin type 2 receptor
ARDS: Acute respiratory distress syndrome
DPP4: Dipeptidyl peptidase 4
PICs: Pro-inflammatory cytokines
MCP-1: Monocyte chemoattractant protein-1
IP-10: Interferon-gamma-inducible protein-10
ALI: Acute lung injury
ALT: Alanine aminotransferase
AST: Aspartate aminotransferase
GGT: Gamma-glutamyl transpeptidase
AKI: Acute kidney injury
CK: Creatine kinase
DIC: Disseminated intravascular coagulation
ACEI: ACE inhibitor
ARB: Angiotensin II receptor blocker
